# Diabetic myonecrosis: a diagnostic challenge in patients with long-standing diabetes

**DOI:** 10.3402/jchimp.v3i1.20494

**Published:** 2013-04-17

**Authors:** Robin Bhasin, Ibrahim Ghobrial

**Affiliations:** Department of Medicine, University of Pittsburgh Medical Center-Mckeesport, Mckeesport PA, USA

**Keywords:** diabetes mellitus, diabetic myonecrosis, proliferative myositis, microvascular complications

## Abstract

A 51-year-old female with a history of type 1 diabetes mellitus (DM) presented with sudden onset of pain and swelling of the left thigh. Her initial evaluation revealed mildly elevated erythrocyte sedimentation rate and creatine phosphokinase. Venous and arterial Doppler studies were negative for DVT and arterial thrombus. Further imaging with CT scan and then MRI revealed an irregular, enhancing space-occupying lesion of the left upper and mid-thigh. Subsequent muscle biopsy showed myonecrosis and proliferative myositis. Both findings are consistent with diabetic myonecrosis, which is a microvascular complication of long-standing poorly controlled DM. The patient was treated with analgesics, supportive care, and optimization of glycemic control. While short-term prognosis is good with adequate healing in a few weeks to several months, long-term prognosis is poor due to underlying extensive vascular disease. Although radiological findings are very suggestive of the diagnosis, most clinicians still need tissue biopsy to rule out other serious conditions such as infections and malignancy.

Diabetic myonecrosis is a rare but serious complication of long-standing diabetes mellitus (DM), commonly associated with diabetic nephropathy and retinopathy. It is caused by infarcted muscle tissue, usually in the thigh. The most common presentation is abrupt onset of unilateral pain, tenderness, and edema.

The first reported case was in 1965 (1). Our literature review included a search of keywords (see above) with a subsequent review of available case reports. Our case is one of the very few reports that include both radiological and histological findings.

## Case report

A 51-year-old female with over 20 years’ history of poorly controlled type 1 DM due to non-compliance, complicated by dialysis-dependent renal failure, retinopathy, and neuropathy presented with sudden onset left thigh swelling and constant severe pain of 7 days duration with no preceding trauma or vascular interventions. She had no fever or recent weight loss.

Her medical history also included coronary artery disease, stroke, hypertension, and mild cirrhosis secondary to hepatitis C.

**Fig. 1:**
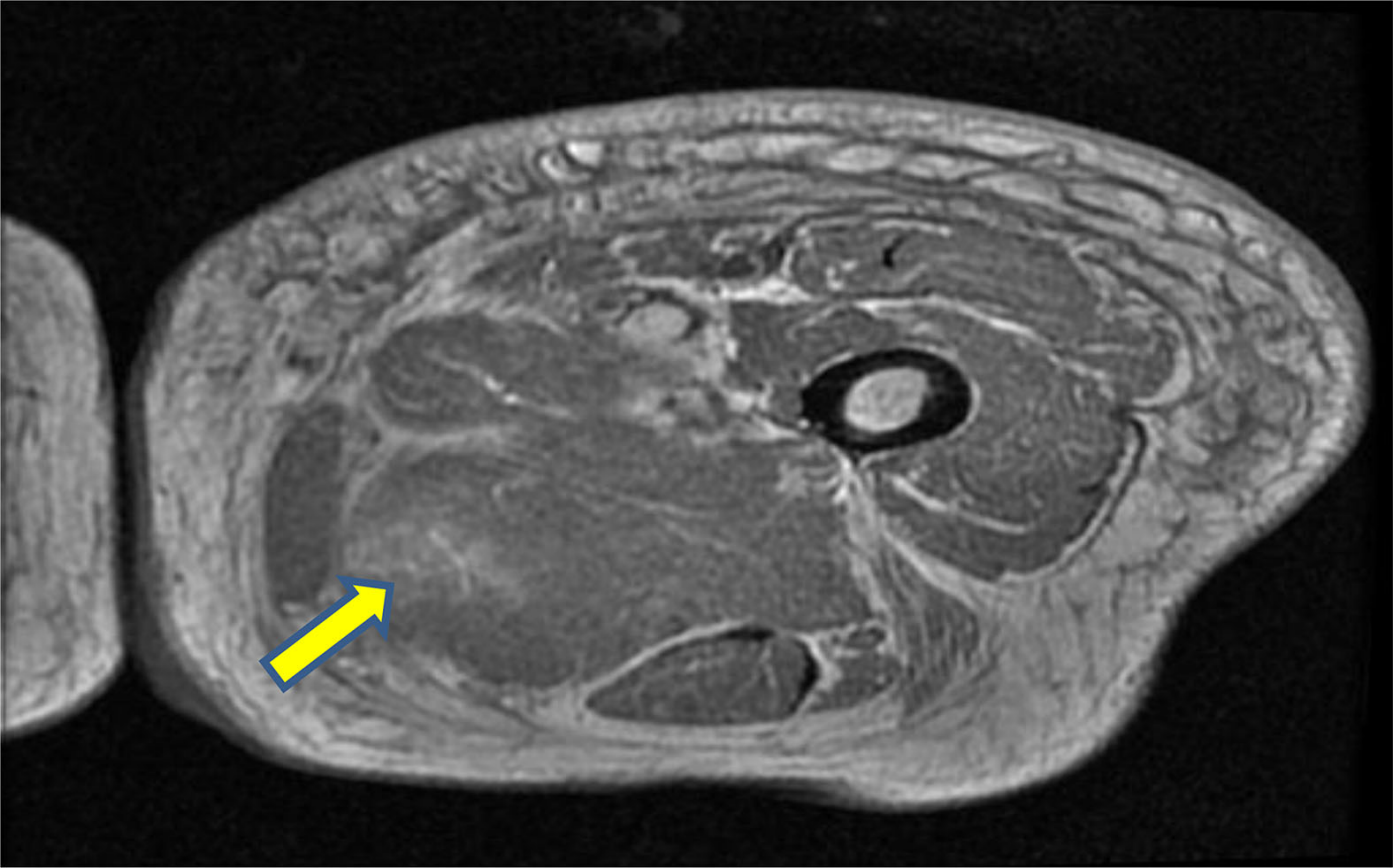
T2-weighted cross-sectional MRI image of the left thigh showing edema and breakdown of muscle architecture.

On examination of her left thigh, she had erythema, edema, and tenderness on the medial aspect. The left thigh was 9 cm larger in circumference than the right. Except for the erythema, overlying skin was normal. She was afebrile. Laboratory work up was significant for WBC of 11,000/dL (normal range 4,000–11,000/dL), mild elevation of erythrocyte sedimentation rate of 45 (normal range<20), C-reactive protein of 1.3 mg/dL (normal range 1–3 mg/dL), and creatine phosphokinase (CPK) of 338 units/L (normal range 38–174 units/L). Plain x-ray did not reveal any gas or calcification. Lower extremity arterial and venous Doppler studies were negative for vascular occlusions. Blood cultures were negative. CT scan of the thigh showed diffuse fat stranding and heterogenous appearance of the left vastus medialis muscle. A magnetic resonance imaging (MRI) with contrast was still needed. Given her renal failure, the patient received hemodialysis immediately after the MRI to mitigate the risk of nephrogenic fibrosing dermopathy (2). The T2-weighted sequence images revealed an irregular, enhancing 20×5 cm space occupying lesion with surrounding edema mostly evident on the medial aspect of the left upper to mid-thigh, involving vastus medialis containing prominent areas of breakdown (Fig. 1). Unfortunately, malignant conditions, specifically soft tissue sarcoma, could not be ruled out by imaging alone, hence a biopsy was still needed. Open muscle was chosen to ensure adequate sampling and to avoid seeding should the lesion turn out to be malignant in nature. An open muscle biopsy revealed homogenous muscle fibers with vacuolation and fragmentation of myofibrils, absence of nuclei, and abundant edema of the interstitial septa. Longitudinal slides showed loss of striation. There was no evidence of infection or neoplasm (Figs. 3, 4, and 5). Also, there was no evidence of uremic calcific arteriopathy (calciphylaxis). The findings were indicative of diabetic myonecrosis.

**Fig. 2:**
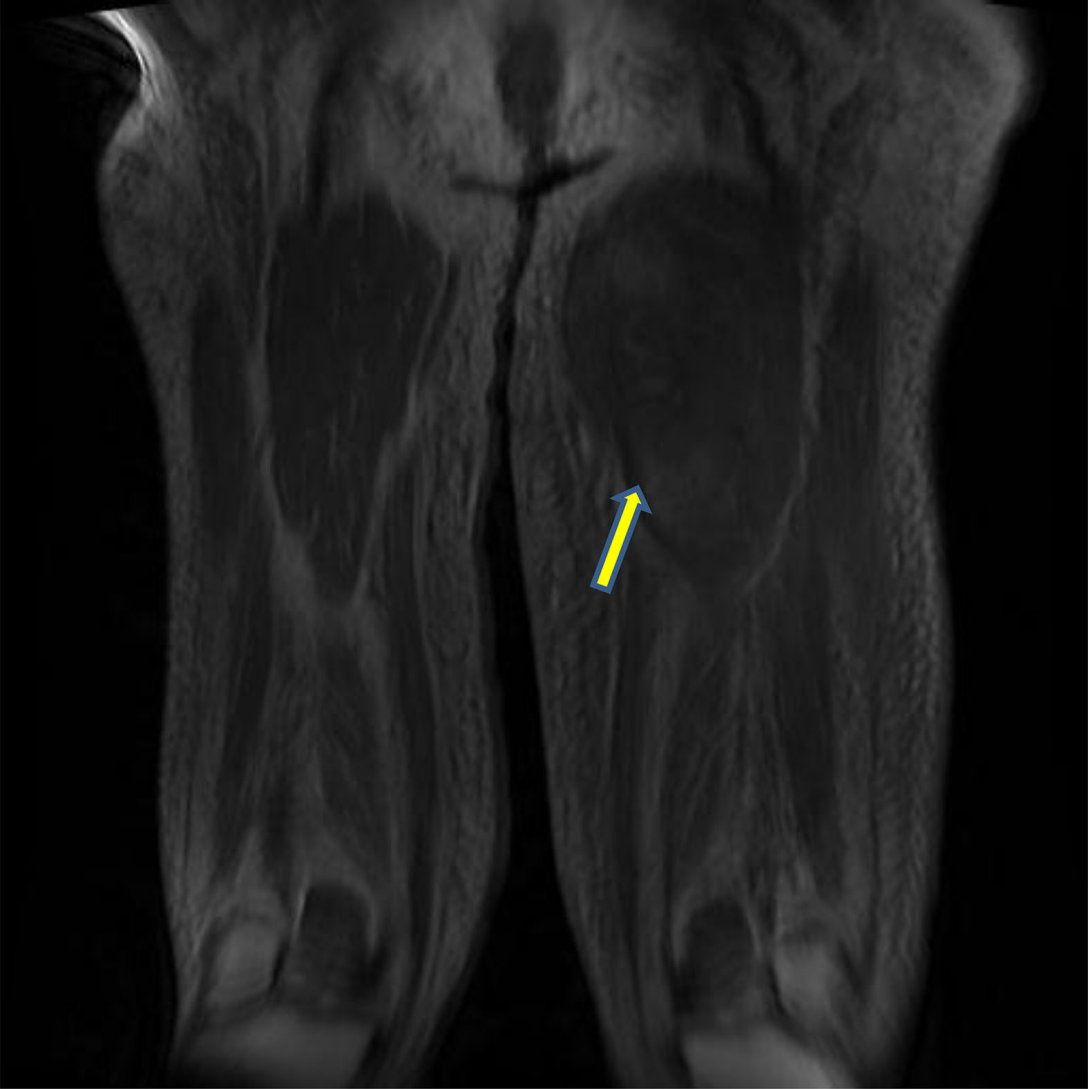
T2-weighted coronal MRI image of the lower extremities showing edema and breakdown of muscle architecture of the left thigh.

**Fig. 3:**
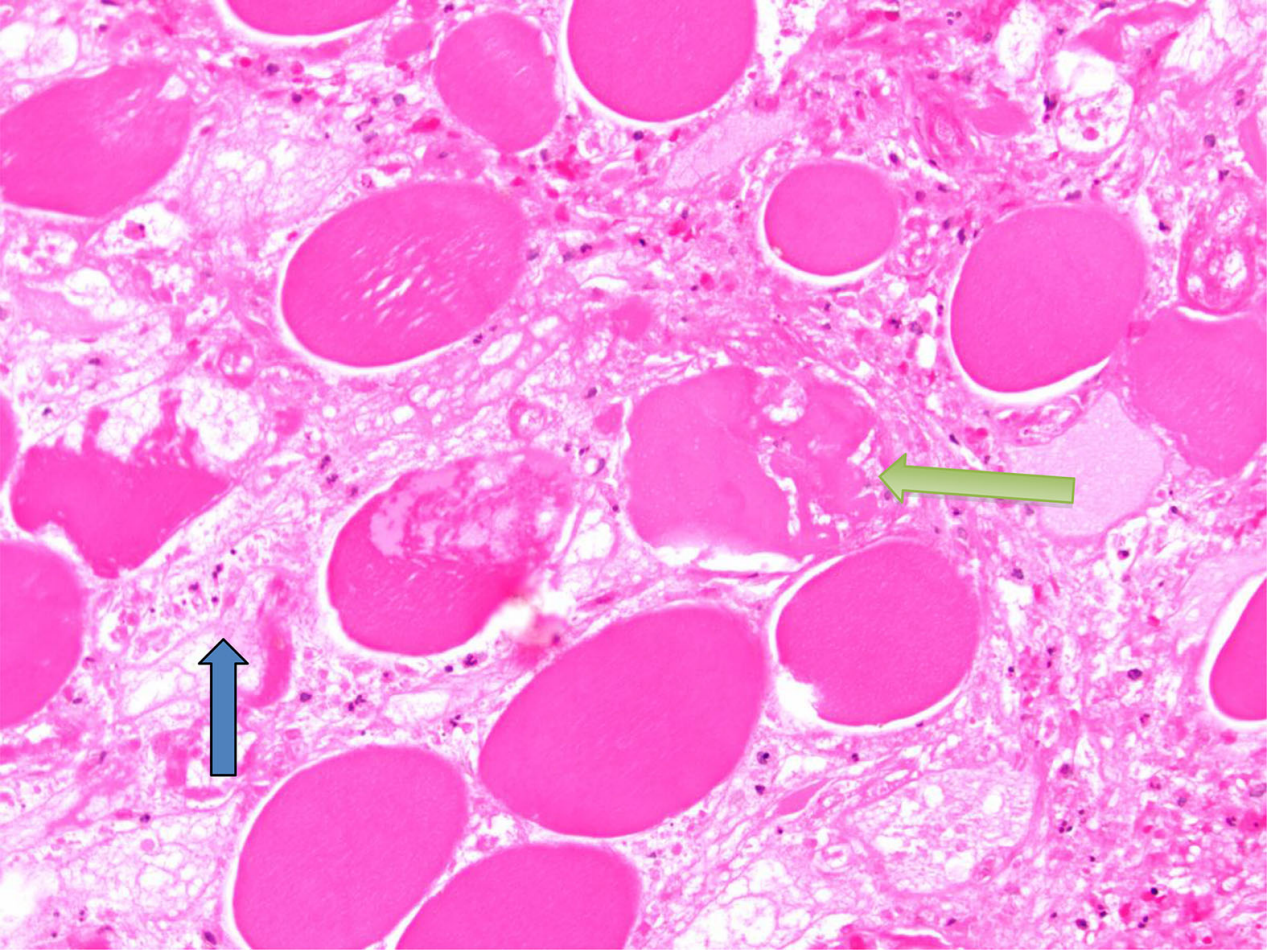
Necrosis of individual muscle fibers (yellow arrow) with edema (blue arrow) in between fibers characteristic of diabetic myonecrosis.

**Fig. 4:**
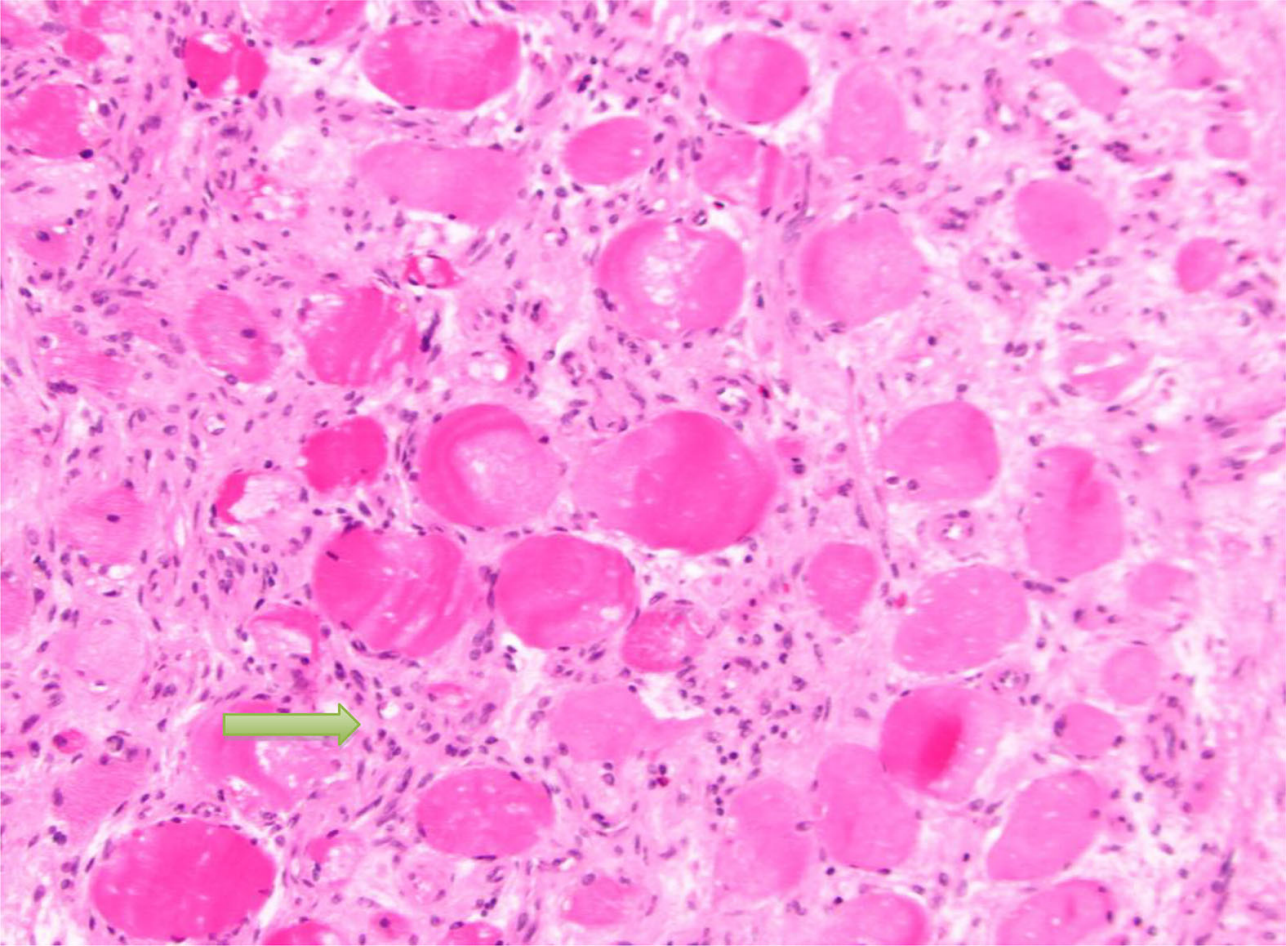
Showing proliferative myositis with expanded myxoid connective tissue with inflammatory cells (yellow arrow). The appearance is also described as checkerboard appearance.

**Fig. 5:**
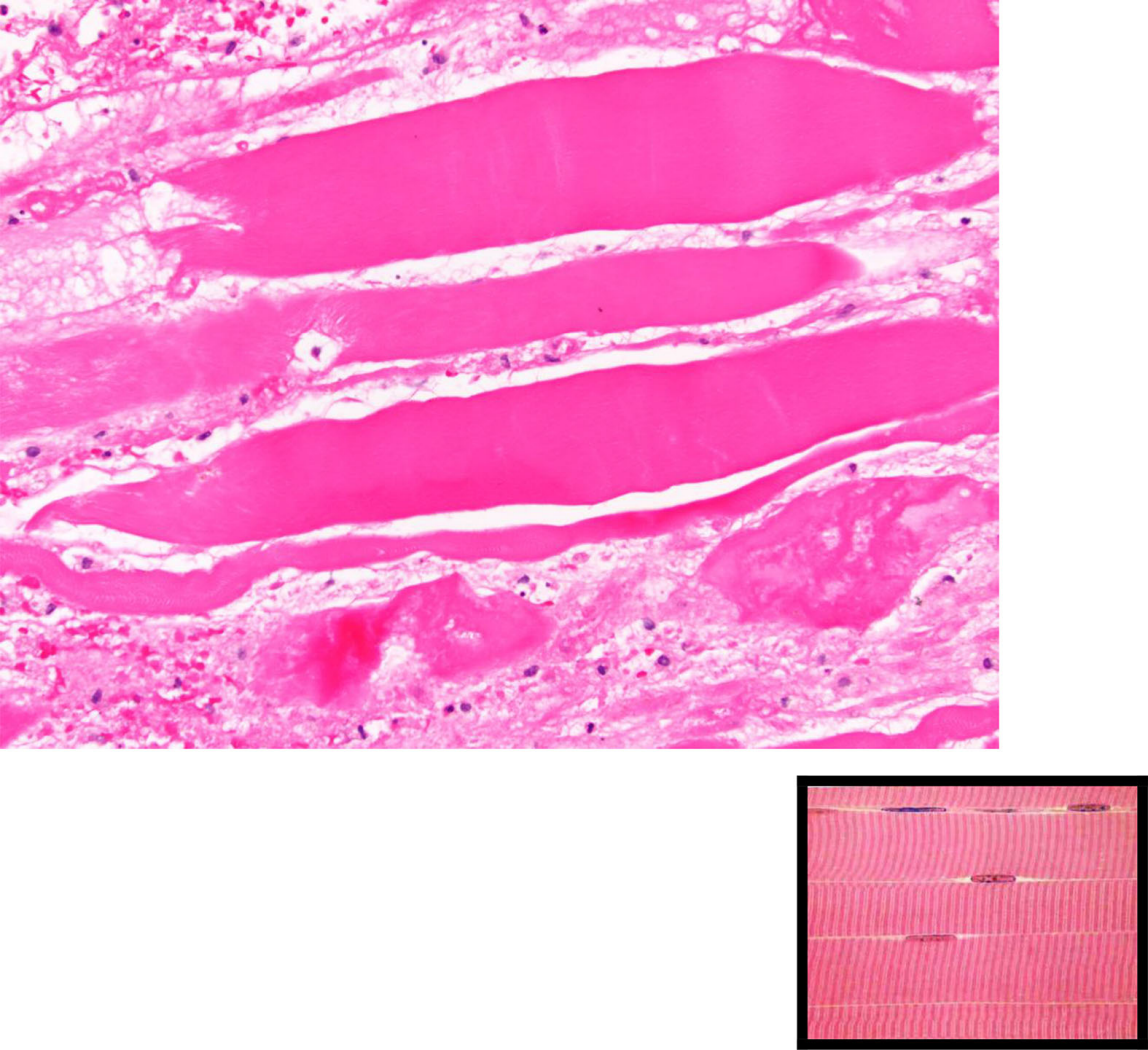
Longitudinal sections showing loss of striation, in comparison to the normal appearance shown in the bottom right corner.

The patient was treated symptomatically with analgesics and bed rest. Inpatient glycemic control was optimized and she became motivated to maintain better glycemic control at home. Her course was complicated by a wound infection at the biopsy site, which necessitated IV antibiotic therapy and was followed by slow healing. The patient sustained significant motor and functional weakness and is currently wheelchair bound.

## Discussion

Diabetic myonecrosis, also known as diabetic muscle infarct, is a rare complication of DM especially in patients with long-standing diabetes (>15 years) with microvascular complications including retinopathy (71%), nephropathy (57%) and/or neuropathy (55%). The average age of presentation is 40 years, and it is more common in women and type 1 DM. Patients commonly present with the abrupt onset of pain, tenderness, and edema of one thigh (3).

Areas most commonly affected include quadriceps (60–65%), hip adductors (13%), hamstrings (8%), and hip flexors (2%) (4). Myonecrosis presents with a tender muscle mass and elevated inflammatory markers and normal or mild elevation of CPK. Differential diagnoses include deep vein thrombosis, hematoma, abscess, pyomyositis, fasciitis, myositis, and malignancy. Imaging with CT can be helpful arriving at a diagnosis based on characteristic findings. Magnetic resonance imaging (MRI) confirms the clinical diagnosis and in some cases of diagnostic uncertainty, a muscle biopsy may be required.

The pathophysiology of diabetic muscle infarction is not well understood. Some authors believe it is secondary to atherosclerosis, diabetic microangiopathy, and ischemia–reperfusion injury (5). Others have hypothesized a role of the coagulation cascade (coagulation–fibrinolysis) causing muscle infarction (6).

Consistent with the findings of our patient, typical findings on MRI include diffuse enlargement of involved muscle groups, partial loss of normal fatty intermuscular septa, and small, focal, rim-enhancing fluid collections. MR imaging using the T1-weighted (isotense) sequence demonstrates a loss of the normal intramuscular septa. However, the degree of involvement of specific muscles is less conspicuous than that with other sequences (7). T2-weighted images (hyperintense) are most likely the best tool to visualize subcutaneous edema, subfascial fluid, and specific muscle involvement (8) (Fig. 2).

Diagnosing diabetic myonecrosis can be challenging. Current recommendations include making the diagnosis on the basis of radiological findings alone and avoidance of open muscle biopsy. Sensitivity of MRI T2-weighted sequence approaches 90% for picking up active muscle disease but with only 43% specificity for muscle infarction (9). Anecdotal evidence suggests that open biopsy prolongs recovery secondary to poor wound healing and also carries risk of complications such as hematoma and infections as was seen in our patient. However, many clinicians resort to biopsy for definitive diagnosis, as this remains the gold standard with specificity approaching 95% (9). Typical findings on a biopsy include: edematous connective tissue and fascia, small pockets of edematous fluid deep to fascia with no pus, but with pale and woody muscle appearance (2, 9). Histologically, abundant lymphocytic infiltration and patchy atrophic fibers with surrounding fibrosis can also be seen (9). Some authors have suggested that using MRI guided biopsy maybe a diagnostic modality since this can help target specific areas of edema and minimize complications (7). Physicians will feel more comfortable making the diagnosis on radiological findings alone if studies confirm concordance between imaging and histological findings. Most cases reviewed have either radiological or histological findings. Our unique case includes both which can be used to demonstrate the aforementioned concordance between radiologic and histological findings.

Delayed wound healing, as mentioned above, is a significant risk for patients who undergo muscle biopsy for definitive diagnosis. This process is related to decreased cellular and growth factor responses, leading to diminished peripheral blood flow and decreased local angiogenesis as seen in other diabetic pathophysiologic processes (10). Other contributing factors include poor macrophagic function, collagen accumulation, quantity of granulation tissue, keratinocyte and fibroblast migration and proliferation, and balance between the accumulation of extracellular matrix components and their remodeling of mixed metalloproteinases (8, 10).

Kapur's three treatment strategies have been described: supportive (bed rest, analgesics), medical (anti-platelet agents, steroids), and surgical exploration treatment modalities (11). The meantime recovery for the three treatment strategies was 5.5, 8.1, and 13 weeks, respectively, making surgery a unfavorable option (6). Hence, the leading treatment modality is supportive. The disease is self-limiting with adequate glycemic control, bed rest, and analgesia.

## Conclusion

Clinicians need to have a high index of suspicion and use sensitive diagnostic tools like MRI (with both T1- and T2-weighted sequences) to make the diagnosis of diabetic myonecrosis. Evidence suggests that the findings reported here could provide clinicians with useful information in making the diagnosis of muscle necrosis without resorting to invasive procedures and that imaging should be the test of choice due to complications of open muscle biopsy. Diabetic myonecrosis has a good short-term prognosis with recovery in 3–4 weeks (sparing complications) but long-term prognosis is poor due to associated vascular complications of DM. Most patients die within 5 years of diagnosis (11).
